# Atypical aetiology of a conjugal fever: autochthonous airport malaria between Paris and French Riviera: a case report

**DOI:** 10.1186/1475-2875-8-202

**Published:** 2009-08-23

**Authors:** Christelle Pomares-Estran, Pascal Delaunay, Annie Mottard, Eric Cua, Pierre-Marie Roger, Bruno Pradines, Daniel Parzy, Hervé Bogreau, Christophe Rogier, Charles Jeannin, Saïd Karch, Didier Fontenille, Dominique Dejour-Salamanca, Fabrice Legros, Pierre Marty

**Affiliations:** 1 Laboratoire de Parasitologie-Mycologie, Centre Hospitalier Universitaire L'Archet, 151 route de Saint Antoine de Ginestière, BP 3079 06202 Nice Cedex 3 France; 2 Laboratoire de Biologie, Hôpital de Fréjus-Saint Raphaël, 83600 Fréjus, France; 3 Infectiologie CHU L'Archet, BP 3079 06202 Nice Cedex 3, France; 4 Institut de Médecine Tropicale du Service de Santé des Armées, 13007 Marseille, France; 5 Entente Interdépartementale de Démoustication Méditerranée, 34184 Montpellier, France; 6 Entomologiste médical, 93600, Aulnay Sous Bois, France; 7 Institut de la Recherche pour le Développement, Caractérisation et contrôle des populations de vecteurs, 34090 Montpellier, France; 8 Département International et Tropical, Institut de Veille Sanitaire, 94415 Saint-Maurice Cedex, France; 9 Centre National de Référence du Paludisme, Université Pierre et Marie Curie, Paris, France

## Abstract

Endemic malaria has been eradicated from France, but some falciparum malaria cases have been described in patients who have never travelled outside the country. Ms. V. 21 year-old and Mr. M. 23 year-old living together in Paris were on holiday in Saint Raphaël (French Riviera). They presented with fever, vertigo and nausea. A blood smear made to control thrombocytopaenia revealed intra-erythrocytic forms of *Plasmodium falciparum*. The parasitaemia level was 0.15% for Ms. V and 3.2% for Mr. M. This couple had no history of blood transfusion or intravenous drug use. They had never travelled outside metropolitan France, but had recently travelled around France: to Saint Mard (close to Paris Charles de Gaulle (CdG) airport), to Barneville plage (in Normandy) and finally to Saint Raphaël. The most probable hypothesis is an infection transmitted in Saint Mard by an imported anopheline mosquito at CdG airport. The DNA analysis of parasites from Ms. V.'s and Mr. M.'s blood revealed identical genotypes. Because it is unlikely that two different anopheline mosquitoes would be infected by exactly the same clones, the two infections must have been caused by the infective bites of the same infected mosquito.

## Background

In a recent paper, Doudier *et al *described two cases of possible autochthonous malaria contracted in Marseille area (southern France) during the summer 2006 [1]. Malaria is a common disease in tropical and sub-tropical areas of over 100 countries, but endemic malaria has been eradicated from France. Nevertheless, some falciparum malaria cases have been described in patients who have never travelled outside France [1-6]. In this case presentation, two autochthonous malaria cases are reported in French Riviera during summer 2008.

## Case presentation

Ms. V. 21 year-old and Mr. M. 23 year-old living together in Paris were on holiday in Saint Raphaël (French Riviera) since of August 13^th^, 2008. In the evening of August, 18^th ^Ms. V. and Mr. M. presented with fever, vertigo and nausea (Day 0). The man's symptoms were more pronounced. He was hospitalized on August 19th in Fréjus-Saint Raphaël Hospital (Day 1). His platelet count was normal (Mr. M. = 208 giga/l). No aetiology was found. The patient went back home the next day with a follow-up but no diagnosis.

On August 22^nd ^(Day 4), his symptoms were still present. The couple returned to the same hospital and a blood cell count, done at that time, found a thrombocytopaenia (Mr. M. = 11 giga/l, Ms. V. = 31 giga/l). A blood smear made to control the thrombocytopaenia and stained by May-Grünwald Giemsa revealed intra-erythrocytic forms of *P. falciparum*. The parasitaemia level was 0.15% for Ms. V and 3.2% for Mr. M. Retrospectively, the Day 1 blood smear of Mr. M. revealed *P. falciparum *with a parasitaemia level of 0.1%. They were hospitalized in Nice University Hospital (French Riviera) and treated with intravenous quinine because of vomiting. Parasitaemia controls done on Day 3 and Day 7 post treatment were negative and clinical evolution was successful.

Parasite DNA was extracted from the blood samples and four *P. falciparum *microsatellite loci (C4M79, Pf2689, Pf2802, TRAP) were genotyped using the method previously described [1]. Length polymorphism analysis revealed that the two infections had exactly the same genotype profile at the four loci and were composed of at least four distinct clones as indicated in Table 1. An *in vitro *drug susceptibility assay was setup on blood samples collected before treatment, for *P. falciparum *drug resistance surveillance [7]. Only the isolate collected before treatment on the woman has an interpretable micro-isotopic test. Inhibitory concentrations 50% (IC_50_) were 2.3 nM, 23.8 nM, 9.1 μM and 2.0 nM for atovaquone, mefloquine, doxycycline and dihydroartemisinin, respectively [7,8]. The isolate was susceptible to all the anti-malarial drugs tested. Unfortunately there weren't enough parasites to realize the test on the second line of antimalarial drug (quinine, chloroquine, lumefantrine and monodesethylamodiaquine). The analysis of resistance molecular markers of the isolates of the two patients revealed a high level of resistance to antifolate drugs (mutations on codons 51, 59 and 108 of the *Pfdhfr-ts *gene, and 436 of the *Pfdhps *gene), the presence of a lysine variant on codon 76 of the *P. falciparum *chloroquine resistance transporter (*Pfcrt*) and no mutation on cytochrome b gene.

**Table 1:** Microsatellite loci genotyping

	C4M79	Pf2689	Pf2802	TRAP
Autochthonous 1*	187, 195, 206, 214	87, 80	133, 141, 148	133, 145
Autochthonous 2†	187, 195, 206, 214	87, 80	133, 141, 148	133, 145
3D7‡	221	87	138	137
W2‡	188	87	146	140

*Patient 1, *Plasmodium falciparum *diagnosed in Ms V.

†Patient 2, *P. falciparum *diagnosed in Mr M.

‡Two parasite strains (3D7 and W2) were genotyped as a positive control; water was used as a negative control.

This couple had no history of blood transfusion or intravenous drug use. They had never travelled outside metropolitan France, however they had travelled around France in the previous fortnight: from the 5^th ^to the 7^th ^to Saint Mard, from the 7^th ^to the 12^th ^to Barneville plage (Normandy) and, finally, from the 13^th ^to the 22^nd ^in Saint Raphaël. Entomological and environmental studies have been conducted in Saint Raphaël and Saint Mard on the 28^th ^of August and the 9^th ^of September. No *Anopheles sp*. mosquito has been collected in these towns. The mean of temperature during their stay in Saint Raphaël and Saint Mard was 23°4 C (30°4 to 17°8 C) and 22°9 C (32°4 to 17°4 C) respectively. However, Saint Mard is 7 kilometers away from International Paris Charles de Gaulle (CdG) airport, where many airport-workers live. Furthermore, a hotel 300 metres away from the couple's home is served daily by a frequent shuttle from CdG Airport. The environmental and climatologic studies showed that there were no international port or airport in the area of Barneville plage and the mean temperature was quite low during their stay (18°5 C). Despite the compatible malaria incubation period no entomological study has been done in that place.

## Conclusion

Autochthonous malaria is defined as *Plasmodium sp. *in blood smears of patients who have never travelled to malaria-endemic areas in the previous 12 months [9]. Transfusion, organ transplantation, intravenous drug use, must also be excluded [9]. The latest case of autochthonous malaria due to *P. falciparum *described in France was in 2006 [1]. Among autochthonous malaria, airport malaria occurs when a *Plasmodium*-infected *Anopheles sp. *mosquito travels from an endemic area to a malaria-free airport [10]. Two cases are described in a couple. The diagnosis was made incidentally on Day 4, when blood smears were done manually for thrombocytopaenia control. In France, it is legally defined that all thrombocytopaenia detected by the analyzer (platelet count below 150 giga/l) have to be controlled. A blood clot has to be excluded and a blood smear is done. On Day 1, in view to the lack of context (no travel history) and of thrombocytopaenia, the diagnosis was not made, but the disease was obviously still ongoing. DNA analysis revealed identical genotypes at all the investigated loci. Because it is unlikely that two different anopheline mosquitoes would be infected by exactly the same four clones, the present data shows that the two infections were caused by the infective bites of the same infected vector specimen, possibly during an interrupted blood feeding. In non-immune individuals most clinical manifestations occur between 7 and 14 days after the infective bite [11]. According to this delay, the potential places of contamination are Saint Mard and Barneville plage and a contamination in Saint Raphäel is excluded. Moreover, 10 cases were described at CdG airport area during the summers 1994 and 1999 [6,10]. In Europe, airport malaria occurred mainly in August when the climate in the vicinity of the origin and destination airport is suitable for the survival of malaria-carrying *Anopheles *[12]. However, an autochthonous anopheline vector infected by a gametocyte carrier in Paris suburb or Barneville plage cannot be excluded [13]. In continental France, nine *Anopheles *species are potential malaria vectors and among them *Anopheles atroparvus *is considered as the primary vector because of its abundance and its anthropophilous potential [14]. Nevertheless, the temperature during their stay in Barneville plage is not in favour of a contamination in this location. In this case, because of incubation period, climate and environmental studies, the most probable hypothesis is an infection acquired during their stay in Saint Mard transmitted by an imported anopheline mosquito at CdG airport.

## Consent

Written informed consent was obtained from the patient for publication of this case report and accompanying images. A copy of the written consent is available for review by the Editor-in-Chief of this journal.

## Competing interests

The authors declare that they have no competing interests.

## Authors' contributions

All authors made substantial contributions to the investigations presented in this manuscript. CP drafted the manuscript and all authors have been involved revising it. All authors read and approved the final version of the manuscript.
